# Short telomeres in short-lived males: what are the molecular and evolutionary causes?

**DOI:** 10.1111/j.1474-9726.2007.00279.x

**Published:** 2007-04-01

**Authors:** Stephanie Jemielity, Masayuki Kimura, Karen M Parker, Joel D Parker, Xiaojian Cao, Abraham Aviv, Laurent Keller

**Affiliations:** 1 Department of Ecology and Evolution, University of Lausanne Lausanne, Switzerland; 2 The Center for Human Development and Aging, University of Medicine and Dentistry of New Jersey, New Jersey Medical School Newark, NJ, USA; 3 School of Biological Sciences, University of Southampton Southampton, UK

**Keywords:** telomere, aging, social insects, disposable soma, telomerase, senescence

## Abstract

Telomere length regulation is an important aspect of cell maintenance in eukaryotes, since shortened telomeres can lead to a number of defects, including impaired cell division. Although telomere length is correlated with lifespan in some bird species, its possible role in aging and lifespan determination is still poorly understood. Here we investigate telomere dynamics (changes in telomere length and attrition rate) and telomerase activity in the ant *Lasius niger*, a species in which different groups of individuals have evolved extraordinarily different lifespans. We found that somatic tissues of the short-lived males had dramatically shorter telomeres than those of the much longer-lived queens and workers. These differences were established early during larval development, most likely through faster telomere shortening in males compared with females. Workers did not, however, have shorter telomeres than the longer-lived queens. We discuss various molecular mechanisms that are likely to cause the observed sex-specific telomere dynamics in ants, including cell division, oxidative stress and telomerase activity. In addition, we discuss the evolutionary causes of such patterns in ants and in other species.

## Introduction

Telomeres, the chromosomes’ ends, are thought to play a key role in determining the replicative potential of vertebrate somatic cells. As somatic cells divide – be it in culture or *in vivo* – their telomeres progressively shorten (Harley *et al*., 1990; Hastie *et al*., 1990; Lindsey *et al*., 1991; Allsopp *et al*., 1992; Counter *et al*., 1992; Zeichner *et al*., 1999; Brümmendorf *et al.*, 2002; Cherif *et al.*, 2003; Haussmann *et al.*, 2003; Hall *et al.*, 2004). Telomere shortening is both due to damage inflicted on the DNA by reactive metabolic by-products, i.e. oxidative stress (von Zglinicki, 2002; Serra *et al.*, 2003), and the fact that DNA polymerase is unable to replicate telomere repeats at the very end of the DNA strand (Olovnikov, 1973; Lingner *et al.*, 1995). Once telomeres are critically short, a signal is relayed to the replicative machinery to stop cell division (Bodnar *et al.*, 1998; Vaziri & Benchimol, 1998; Yang *et al.*, 1999). This irreversible cell-cycle arrest is referred to as replicative senescence or telomere-induced cellular senescence (Campisi *et al.*, 2001; Wright & Shay, 2002). In contrast to somatic cells, germ-line cells divide indefinitely as they are passed across generations and are not subject to telomere shortening (Allsopp *et al.*, 1992; Coviello-McLaughlin & Prowse, 1997), most likely because germ-line cells express telomerase at a higher level than adult somatic tissues (Kim *et al.*, 1994; Taylor & Delany, 2000). This enzyme, a reverse transcriptase, adds back telomere repeats onto the ends of chromosomes and thus counteracts telomere attrition (Greider, 1990).

Blood-cell telomere length is associated with survival in two bird species (Haussmann *et al.*, 2005; Pauliny *et al.*, 2006) and telomere length in peripheral leukocytes may be correlated with mortality in elderly humans (Cawthon *et al.*, 2003, but see Martin-Ruiz *et al.*, 2005). But the question of how and under what circumstances telomere dynamics and replicative senescence are tied to organismal lifespan is still unsolved. To answer this question, telomere biology needs to be studied at all possible levels – from cells to organisms to evolutionary factors (Monaghan & Haussmann, 2006).

Especially at the organismal level, comparative data are still scarce and largely limited to vertebrates. This makes it difficult to predict what evolutionary factors influence telomere length regulation. For instance, it is unclear whether at the intraspecies level different life-history strategies are associated with differences in telomere length and how and why different telomere length patterns are established. We tackle these questions by investigating telomere dynamics in queens (reproductive females), workers (nonreproductive females) and males of the ant *Lasius niger*, an insect species that has previously been shown to possess TTAGG-type telomere repeats (Lorite *et al.*, 2002). Ant queens, workers and males have evolved a tremendous difference in lifespan (Hölldobler & Wilson, 1990; Keller & Genoud, 1997) and other life-history traits. In *L. niger*, for instance, queens can reach the astonishing age of 28 years (Hölldobler & Wilson, 1990), whereas workers typically die within a few years and males within 2 to 3 months (Parker *et al.*, 2004; S. Jemielity & L. Keller, unpublished data). Importantly, these differences in lifespan among castes cannot be due to caste-specific alleles or chromosomes: queens and workers generally develop from identical diploid eggs (Wheeler, 1986; Hölldobler & Wilson, 1990) and males differ from queens and workers only insofar as males are initially haploid (Hölldobler & Wilson, 1990; for an overview of ant development see Experimental procedures).

## Results and discussion

### Telomere length differs between male and female ants

**Fig. 1:**
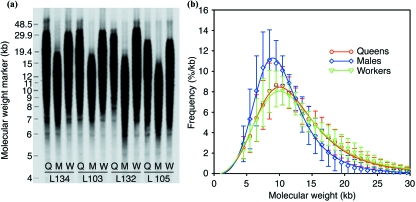
Telomere length comparison among somatic tissues of adult queens, males and workers. (a) Typical terminal restriction fragment (TRF) Southern blot. TRFs are from heads and thoraces of 1- to 3-day-old queens (Q), males (M) and workers (W). Four out of nine nests (L134, L103, L132 and L105) are depicted. The mean TRF length calculated from such Southern blots was used as a measure of telomere length. The figure shows that TRF length was considerably shorter in males than in queens and workers. (b) TRF length distribution in queen, male and worker somatic tissues of eight nests. One nest (L128), which had produced only males and workers, but no queens, was excluded from the analysis. Error bars represent standard deviation. The lines are four-parameter logistic dose–response peak curves fitted to average frequencies for each caste (for technical details, see Kimura *et al.* submitted). The figure suggests that the mean telomere length of males is shorter because short telomeres are over-represented and long telomeres are under-represented in males compared to queens and workers.

To test whether telomere length is associated with caste-specific lifespan, we measured the mean terminal restriction fragment (TRF) length in somatic tissues (heads and thoraces) of 1- to 3-day-old adult queens, workers and males from nine different nests. Although we found strong inter-caste variation in telomere length (Table 1, Fig. 1a), telomere length was only partially tied to caste-specific lifespan. On average, the mean TRF length in male somatic tissues was significantly shorter than in queen (Wilcoxon signed rank test: *n* = 8, *P* = 0.02) and worker somatic tissues (Wilcoxon signed rank test: *n* = 9, *P* = 0.01). The short-lived males had telomeres that were on average 2.1 kb (16.2%) and 2.3 kb (17.4%) smaller than those of the much longer-lived queens and workers, respectively (Table 1). There was, however, no positive relationship between lifespan and telomere length in the two female castes. Workers even tended to have slightly (0.2 kb, 1.5%) longer telomeres than the longer-lived queens. [This difference was significant based on mean TRF length analysis (Wilcoxon signed rank test: *n* = 8, *P* = 0.04), but not based on median TRF length analysis (Wilcoxon signed rank test: *n* = 8, *P* = 0.6).]

**Table 1:** Mean TRF length (kb) of heads and thoraces from 1- to 3-day-old adult queens, males and workers

Nest	L42	L51	L103	L105	L108	L122	L128	L132	L134	Mean ± SD
Queens	15.13	12.50	13.52	12.31	13.16	10.18	*	13.70	13.34	13.0 ± 1.4
Males	14.72	9.17	11.11	9.51	11.19	10.86	11.11	9.79	10.77	10.9 ± 1.6
Workers	14.76	12.90	14.32	12.18	13.57	10.92	12.55	14.14	13.90	13.2 ± 1.2

* There is no measurement for queens from nest L128 because this nest produced only males and workers.

Interestingly, the difference in mean telomere length between males and queens/workers is mainly due to the over-representation of short telomeres and under-representation of long telomeres in males relative to queens and workers (Fig. 1b): Both skewness and kurtosis values of male TRF distributions were significantly greater than those of queen and worker TRF distributions (anova with post-hoc Duncan's multiple tests, d.f. = 14, *P* < 0.05 in all cases). This difference in distribution shape is consistent with the idea that by the time they are adults, males have accumulated more short telomeres than queens and workers. In contrast, queen and worker TRF distributions did not differ significantly in terms of skewness and kurtosis.

To rule out the possibility that the difference in telomere length between males and queens/workers might simply reflect sex-specific differences in the tissues that compose the body-parts analyzed, we tested whether tissue composition had an influence on mean TRF length. This was done by comparing mean TRF length of queen heads, which are mainly made of gland and nerve tissues, to that of queen thoraces, which are mainly composed of flight muscle and adipose tissue. In both samples analyzed, head and thorax mean TRF length differed by less than 0.2 kb. In addition, mean TRF length in heads and thoraces of 1- to 3-day-old workers was very similar to that measured in worker gasters (i.e. the principal part of the abdomen; data not shown), supporting the view that the difference in telomere length between males and queens/workers does not stem from differences in tissue composition.

Longer telomeres in females vs. males were also discovered in four mammalian species. Leukocytes from adult women (Jeanclos *et al.*, 2000; Benetos *et al.*, 2001; Nawrot *et al.*, 2004) and somatic cells from adult female rats (Cherif *et al.*, 2003) had significantly longer telomeres than the corresponding male cells. Incidentally, in both these species females are the longer-lived sex. In the mouse *Mus spretus* (Coviello-McLaughlin & Prowse, 1997) and in the rhesus monkey, *Macaca mulatta* (A. Aviv, unpublished data), adult females also showed also longer telomeres than adult males. But whether females are significantly longer-lived in these species is unknown.

### When and how are the sex-specific telomere length patterns established?

**Fig. 2:**
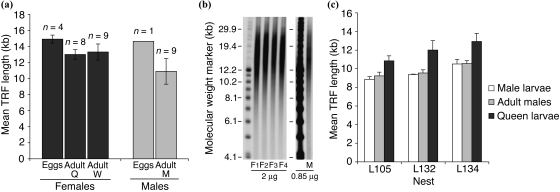
Telomere dynamics during queen, male and worker development. (a) Mean TRF length in female and male eggs vs. adult somatic tissues. Depicted are mean and standard deviation. Sample sizes are given above the bars. This figure shows that telomeres shorten during female and male development (Q, queen; W, worker; M, male). (b) TRF blot of 1- to 10-day-old male (i.e. unfertilized) eggs vs. female (i.e. fertilized) eggs. All samples are pools of eggs from several queens. Because of the smaller amount of DNA, the signal of the male egg sample (M) was generated using a longer exposure of the blot. Telomere length in male eggs is similar to that in female eggs (F1–4). (c) Mean TRF length of queen and male larvae and young adult males. Depicted are mean and standard deviation of two independent measurements. The results show that telomere length differs considerably between young male and queen larvae, and that male telomeres do not shorten between the larval stage tested and early adulthood.

The sex-specific differences in telomere length observed in ants most likely arise through faster telomere attrition in males compared to queens and workers. The mean TRF length of 1- to 3-day-old female eggs was significantly longer than that in somatic tissues of adult queens (Wilcoxon rank sum test: *n* = 4, *m* = 8, *P* = 0.02) and workers (Wilcoxon rank sum test: *n* = 4, *m* = 9, *P* = 0.01), suggesting that telomeres shorten during queen and worker development (Fig. 2a). Telomeres also appear to shorten during male development (Fig. 2a), but unfortunately we could not test this statistically, as it was impossible to obtain male eggs for more than one telomere length measurement. Nevertheless, since the mean TRF length and TRF length range of this male egg replicate was similar to that in female eggs (mean TRF length = 14.6 kb in males vs. 14.9 ± 0.5 kb in females; Fig. 2b), faster telomere shortening in males is likely to occur.

Accelerated telomere shortening in males has also been suggested to account for sex-specific telomere length patterns in rats and humans (Okuda *et al.*, 2002; Cherif *et al.*, 2003). However, in contrast to mammals, where these patterns are established in the course of extra-uterine life (Okuda *et al.*, 2002; Cherif *et al.*, 2003), telomere length divergence appears to already have taken place during early larval development in ants. First, a pronounced difference in telomere length was already present between young male and queen larvae (Fig. 2c). In the three nests analyzed, the mean TRF length of queen larvae was on average 2.3 ± 0.3 kb longer than that of male larvae. Second, in males, mean TRF length did not change between the larval stage investigated and early adulthood (Fig. 2c).

### Telomeres are longer in male germ-line tissues than in somatic tissues

**Fig. 3:**
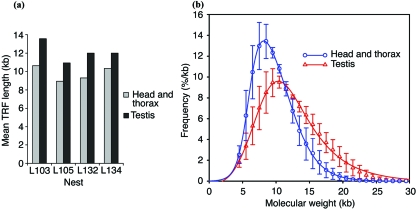
Telomere length comparison between male somatic and germ-line tissues. (a) Mean TRF length in somatic and germ-line tissues of 1- to 3-day-old males. The mean TRF length of testes is considerably greater than that of heads and thoraces. (b) Mean TRF distribution in male somatic tissues and testes from four nests. The lines are four-parameter logistic dose–response peak curves fitted to average frequencies for each tissue type (for technical details see Kimura *et al.* submitted). The mean telomere length in testes is greater than that in somatic tissues because in testes long telomeres are over-represented and short telomeres are under-represented compared to somatic tissues.

To test whether short telomeres are characteristic for male tissues in general, we compared mean TRF length in testes and somatic tissues (head and thorax) of 1- to 3-day-old adult males from four nests. In all four replicates the mean TRF length of testes was considerably greater than the mean TRF length of somatic tissues (Fig. 3a), suggesting that males do have the ability to maintain telomere length at a higher level than that observed in somatic tissues. On average, the mean TRF length of testes was 2.3 ± 0.6 kb (23 ± 6%) greater than the mean TRF length of somatic tissues, which is comparable to the difference in telomere length found between sperm (or testes) and somatic tissues in several vertebrate species: 14% in cattle (Kozik *et al.*, 1998), 24% in pigs (Kozik *et al.*, 1998), and 11–21% in the mouse *Mus spretus* (calculated from Coviello-McLaughlin & Prowse, 1997). Only in humans does the germ-line-vs.-soma difference in telomere length seem to be somewhat greater (∼39% as calculated from de Lange *et al.*, 1990; 69% according to Kozik *et al.*, 1998).

In ants the greater mean telomere length in testes was most likely due to the over-representation of long telomeres and the under-representation of short telomeres in testes compared to somatic tissues (Fig. 3b): all four testes samples had considerably lower skewness and kurtosis values than the four head and thorax samples (data not shown). Thus, male germ-line tissues seem to accumulate fewer short telomeres than male somatic tissues.

### Proximate causes of the sex-specific telomere length patterns

At the proximate (i.e. molecular) level several more or less plausible factors can explain the sex-specific telomere length patterns observed in somatic tissues. First, male telomeres could be shorter simply because male cells undergo more cell divisions than female cells. This is, however, unlikely because *L. niger* males are six to eight times lighter than queens (range based on fresh weights of 1- to 3-day-old adults from five nests). Second, one might argue that ant males have shorter telomeres because they develop from haploid eggs, whereas females develop from diploid eggs. But both male and female eggs were similar in telomere length, suggesting that the ploidy level *per se* is not a plausible explanation. In addition, the truly haploid phase in male ants is limited to a short period of time, suggesting that any indirect effect of the ploidy level on telomere length (e.g. via cell size) should be small. Ant males and males from other hymenopteran species at least partially restore the ploidy level of their somatic tissues to that found in the corresponding female tissues (Merriam & Ris, 1954; Risler, 1954; Rasch *et al.*, 1977; Aron *et al.*, 2005). This endoreduplication process is thought to be necessary for dosage compensation and starts before or within hours after the first larval instar hatches (Mittwoch *et al.*, 1966; Rasch *et al.*, 1977).

A more plausible proximate explanation is that male somatic tissues have shorter telomeres because they are more subject to oxidative stress. Oxidative stress is known to affect telomere length in proliferating mammalian cells (von Zglinicki, 2002; Serra *et al.*, 2003), probably by causing single-strand breaks in telomeric DNA (von Zglinicki *et al.*, 2000). Ant males might produce more oxidative-stress-inducing metabolic by-products than females or they may have a less efficient defense system against these molecules. Another possible explanation for the shorter male telomeres is that males and females have different levels of a hormone that directly affects telomere dynamics. For instance, the hormone estrogen may have an influence on telomere dynamics in mammalian cells (Kyo *et al.*, 1999; Lee *et al.*, 2005).

**Fig. 4:**
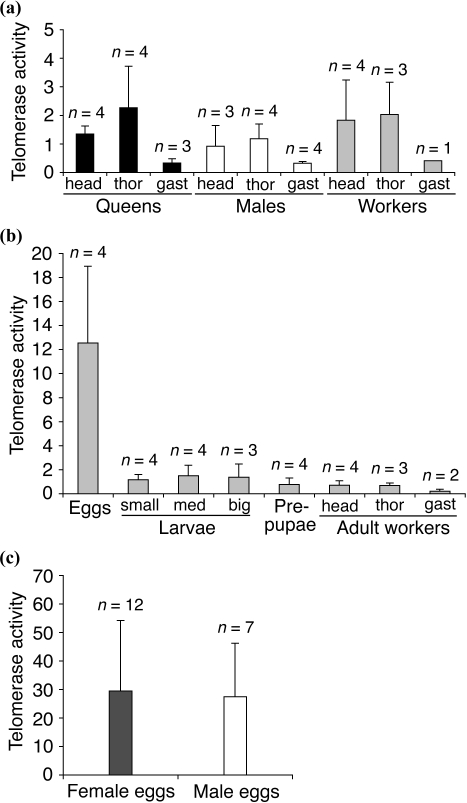
Telomerase activity measurements. (a) Depicted are the mean TRAP assay values and standard deviations of 1- to 3-day-old adult queens, males and workers. Sample sizes are given above the bars. In adult ants telomerase is active in all body sections, i.e. also in those consisting of purely somatic tissues. Telomerase activity is, however, low in all samples, with no pronounced differences among castes (Thor, thorax; gast, gaster). (b) TRAP assay measurements performed on various worker developmental stages. Depicted are the mean and standard deviation of two to four independent samples. Telomerase activity appears to be the highest at the egg stage (med, medium; thor, thorax; gast, gaster). (c) Mean TRAP assay values and standard deviations for 1- to 3-day-old female and male eggs. Sample sizes are given above the bars. Telomerase activity is similar in male and female eggs and appears to be highly variable in both groups.

Finally, male telomeres could be shorter than female telomeres because telomerase is inactive or less active in male somatic cells. To test this hypothesis we carried out quantitative TRAP assays. According to these assays telomerase was active in all body parts of *L. niger* adults (Fig. 4a), as well as during all worker developmental stages (Fig. 4b). We found no evidence for a link between telomerase activity and sex-specific telomere length. Although telomerase tended to be slightly less active in adult males than in adult queens and workers (Fig. 4a), this pattern was not observed in all four replicates (data not shown). We could also find no significant difference in telomerase activity between 1- to 3-day-old male and female eggs (Fig. 4c; *t*-test: *t* = 0.2, d.f. = 17, *P* = 0.85). However, the lack of evidence for sex-specific telomerase activity does not completely exclude the possibility that telomerase is involved in creating the sex-specific telomere length patterns observed. The access of telomerase to telomeres is regulated by several telomere-binding proteins (Blackburn, 2000), and thus equal telomerase activity might not necessarily imply an equal effect on telomeres.

### Ultimate or evolutionary causes for sex-specific telomere length patterns

It has been suggested that telomere shortening has evolved as a protection mechanism against cancer (Wright & Shay, 2001; Campisi, 2003). But in the case of ants it seems unlikely that the small, ephemeral males would have to have a more powerful protection against cancer than the much larger and longer-lived queens. A better evolutionary explanation might be provided by the disposable soma theory. This theory posits that organisms modulate their investment in the maintenance and repair of the soma according to their life-history strategy, which, in turn, is primarily shaped by the level of extrinsic mortality experienced. Organisms that face a high level of extrinsic mortality should be selected to invest more in early reproduction and less in somatic maintenance, which comes at the cost of a shorter intrinsic, i.e. environment-independent lifespan (Kirkwood, 1977,^1981^; Kirkwood & Rose, 1991). Accordingly, within a given species the shorter-lived sex or caste is predicted to invest less in somatic maintenance, one aspect of which may be telomere length maintenance. Importantly, this does not imply that telomere shortening substantially contributes to determining lifespan. From an evolutionary perspective this would be unlikely, as many genes and molecular pathways are thought to affect somatic maintenance (Kirkwood & Rose, 1991). Incidentally, the disposable soma theory also predicts that an organism should invest more resources in the maintenance of telomeres in its germ-line cells than in its ‘short-lived’ somatic cells (Kirkwood, 2005).

Our finding that the short-lived males have shorter telomeres than the longer-lived queens and workers fits the predictions of the disposable soma theory. Previously published data on sex-specific telomere dynamics in humans (Jeanclos *et al.*, 2000; Benetos *et al.*, 2001; Nawrot *et al.*, 2004) and in rats (Cherif *et al.*, 2003) are also in line with these predictions. By contrast, some data appear at odds with the disposable soma theory. Somatic tissues of workers did not have shorter telomeres than those of the much longer-lived queens. Likewise, telomere length was not correlated with lifespan across several strains of the nematode *Caenorhabditis elegans* (Raices *et al.*, 2005). However, it is possible that these incongruent data can be explained by constraints that disrupt the correlation between telomere length and lifespan. For instance, queens and workers may have similar telomere lengths as a result of their shared developmental history: because they develop from a bipotential developmental stage.

Another explanation for sex-specific telomere length patterns is that female and male telomere dynamics may be differentially constrained, i.e. telomere length can only evolve within sex-specific fixed limits. Such limits, which would be independent of lifespan, could for instance be imposed by sex-specific trade-offs mediated by hormones, gene expression patterns or other traits that differ between males and females. While this explanation is consistent with the sex-specific telomere length patterns observed in somatic tissues of adult ants, humans and rats, it leaves us with the puzzle of why germ-line-tissues but not somatic tissues are able to overcome these constraints.

## Conclusions

To our knowledge this is the first extensive study on telomere dynamics in an insect species. We show that telomere dynamics in ants are similar to those in mammals: telomeres shorten considerably during development, the rate of shortening differs between males and females and germ-line tissues have longer telomeres than somatic tissues. This similarity is remarkable, given the fundamental differences in development and sex determination between mammals and ants. The only differences that we found were quantitative in nature. In ants telomere length divergence between sexes seems to start earlier and the difference in lifespan and telomere length between males and females is more pronounced than in mammals. Based on our findings and similar data from the literature, we provide two plausible explanations for sex-specific telomere length patterns. To decide which of these explanations is better, further comparative studies are needed, especially from species in which males are the longer-lived sex. In such species males would be expected to have longer telomeres than females if the disposable soma interpretation holds, whereas the opposite would be expected according to the sex-specific-constraint hypothesis. Finally, our finding that queens and workers had telomeres of similar length suggests that telomere length is not among the factors that cause the almost tenfold difference in lifespan between these two castes.

## Experimental procedures

### *Lasius niger* ants

**Fig. 5:**
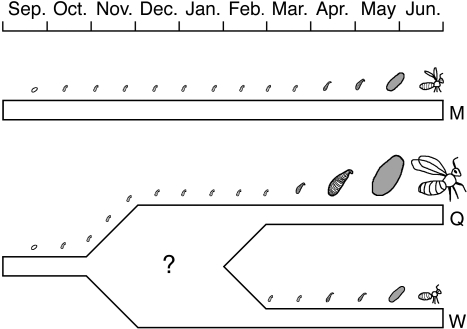
Timeline of *L. niger* development. Eggs, larvae and adults are drawn to scale. Male and female eggs are laid between the end of September and the beginning of October. After about 2 weeks the larvae, which resemble tiny hooks, hatch and start overwintering. The main larval growth period is in the following spring, between the end of March and the end of May. After this growth phase larvae spin a cocoon in which they undergo metamorphosis within an estimated two to three weeks. There is a second phase of worker production in fall, which was not included in this timeline because it is not relevant to our study. The question mark indicates that it is unknown when the queen and worker developmental pathways diverge (M, male; Q, queen; W, worker).

Most ant species, including *L. niger*, are characterized by a haplo-diploid sex-determination system. Unfertilized haploid eggs develop into males, whereas fertilized diploid eggs develop into either of two types of females: queens or workers (Hölldobler & Wilson, 1990). Whether a female egg or larva enters the queen or worker developmental pathway is generally not determined genetically. It depends on the environmental conditions experienced, such as temperature or the quality of the food received, which activates different gene expression patterns (Wheeler, 1986; Hölldobler & Wilson, 1990). A comparative timeline for the development and growth of *L. niger* queens, males and workers is given in Fig. 5. This figure is based on published and unpublished data from Jemielity & Keller (2003).

### Sampling and dissections

In order to obtain age-matched adults for TRF length analysis, we collected queen, male and worker pupae, as well as a few hundred adult workers from nine *L. niger* field nests in early June 2004 and transferred them to plastic boxes in the laboratory. Within 1–3 days after they emerged from the cocoon, young males, queens and workers were frozen in liquid nitrogen. Prior to DNA extraction, the ants were dissected on dry ice to separate heads and thoraces (purely somatic tissues) from gasters (somatic and germ-line tissues). Testes were dissected out from thawed male gasters. To obtain enough DNA, we pooled body sections or tissues of at least 10 nest-mates per sample.

Larvae and eggs used for TRF length measurements were sampled as follows: Male and queen larvae were gathered from three field nests in April 2004 and were directly frozen in liquid nitrogen. At that time male larvae weighed less than 20% of their final weight, while queen larvae had reached about 50% of their final weight. Dissections confirmed that both queen and male larvae still lacked differentiated germ-line tissues (i.e. they essentially consisted of somatic tissues). All larval samples were pools of at least seven nest-mates. Each of the four sets of female eggs analyzed was obtained by pooling hundreds of 1- to 10-day-old eggs from 16 newly mated queens. Such queens produce only female (fertilized) eggs for several years. We also obtained 1- to 10-day-old male (unfertilized) eggs from 60 virgin queens, but since these queens produce only very few eggs, all male eggs had to be pooled into a single sample.

Adult ants, worker brood and eggs used for telomerase activity assays were sampled in the following way. Freshly frozen 1- to 3-day-old adult queens, males and workers from four nests were dissected into heads, thoraces and gasters on dry ice. Each sample was a pool of body sections of at least 10 nest-mates. We also prepared four independent sets of various worker developmental stages: eggs of mixed age, small larvae, medium larvae, big larvae, pre-pupae and adult workers. This was done by pooling several individuals from three laboratory nests per set. Finally, we measured telomerase activity in seven independent sets of 1- to 3-day-old male eggs (each obtained by pooling eggs from several virgin field queens) and 12 independent sets of 1- to 3-day-old female eggs (each obtained by pooling eggs from several newly mated field queens).

### DNA extractions and TRF length measurements

Genomic DNA was isolated using Qiagen (Hombrechtikon, Switzerland) Genomic-tip columns. A 0.5-µg aliquot of all samples was analyzed by pulsed-field-gel electrophoresis (Chef-DR III, Bio-Rad, Reinach, Switzerland) to confirm that the DNA was intact. Its mean molecular size was 77 ± 7 kb for all samples, except for the three queen larva samples where it was 31 ± 3 kb. DNA samples (2 µg unless stated otherwise) were digested overnight with restriction enzymes *Hinf*I (0.5 U µL^−1^) and *Rsa*I (0.5 U µL^−1^) (Roche, Rotkreuz, Switzerland). Samples and DNA markers (1-kb DNA ladder and High Molecular Weight DNA Marker, Invitrogen, Carlsbad, CA, USA) were resolved on a 0.3% agarose gel (20 cm × 20 cm) at 50 V (GNA-200, Pharmacia Biotech, Piscataway, NJ, USA). After 16 h, the DNA was depurinated for 15 min in 0.25 N HCl, denatured 30 min in 0.5 m NaOH/1.5 m NaCl and neutralized for 30 min in 0.5 m Tris, pH 8/1.5 m NaCl. The DNA was transferred for 1 h to a positively charged nylon membrane (Roche) using a vacuum blotter (Boeckel Scientific, Hamburg, Germany). The membranes were hybridized overnight at 65 °C with the telomeric probe [digoxigenin 3′-end labeled 5′-(CCTAA)_3_] in 5× SSC, 0.1% Sarkosyl, 0.02% SDS and 1% blocking reagent (Roche). The membranes were washed at room temperature as follows: three times 15 min in 2× SSC, 0.1% SDS and once 15 min in 2× SSC. The digoxigenin-labeled probe was detected by the digoxigenin luminescent detection procedure (Roche) and exposed on X-ray film. The DNA markers were detected by random-primed, digoxigenin-labeled probes. We scanned all autoradiographs with a computing densitometer (model 300 A, Molecular Dynamics, Sunnyvale, CA, USA) and digitized the TRF signal with Image Quant (version 3.3, Molecular Dynamics). Using the program SAS (version 9.1) we then calculated for each sample the mean TRF length between molecular weights of 4–50 kb: 
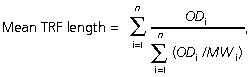
 where *OD_i_* is the background-adjusted optical density at a given position in the lane and *MW_i_* is the molecular weight at that position. Using SAS we also computed the median TRF length, as well as the skewness and kurtosis of the TRF distribution of each sample. Skewness measures the asymmetry of a distribution, while kurtosis is a measure of the proportion of elements near the center of the distribution compared to that of the distribution's tails.

### Telomerase activity measurements

**Fig. 6:**
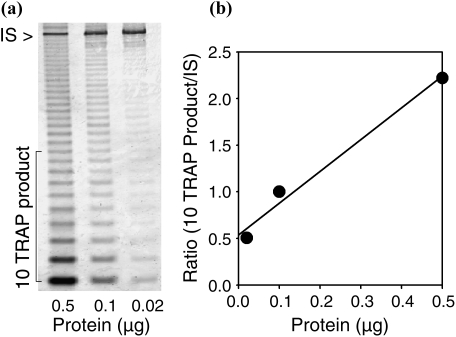
Illustration of the method to quantitate telomerase activity. (a) Depicted are TRAP assay products at three concentrations of the control protein extract, which was derived from somatic tissues of queens from one nest. (b) This panel shows the ratios of optical density at the three concentrations for the first 10 bands/internal standard (IS).

Telomerase activity was measured by Telomeric Repeat Amplification Protocol (TRAP) assays. The TRAP assay for insects (Sasaki & Fujiwara, 2000) was modified to detect ant telomerase activity quantitatively (Fig. 6). The reaction mixture (50 µL) consisted of 0.1 µg protein extract, 0.2 mm dNTPs, 1 µm TS primer (5′-AATCCGTCGAGCAGAGTT), 1 µm BmCXa primer (5′-GTGTAACCTAACCTAACC), 100 attogram internal standard, 0.005% BSA, 1× reaction buffer, and 0.2 U *Taq* polymerase (Roche). After incubating the reaction tubes at 30 °C for 30 min, 32 cycles of two-step PCR were performed at 94 °C/30 s and 59 °C/30 s. The PCR products were resolved on 10% nondenaturing polyacrylamide gels, stained with SYBR green I and scanned by typhoon (Amersham Biosciences, Piscataway, NJ, USA). To be able to compare samples across gels and to verify that the approach was quantitative, on each gel, TRAP assay products of 0.02, 0.1, and 0.5 µg of a control protein extract (derived from somatic tissues of queens from one nest) were loaded (Fig. 6). For each sample the ratio of intensity of the first 10 telomerase ladder bands to the internal standard band was calculated and telomerase activity was expressed as relative units compared to the 0.1 µg control protein extract. Samples that yielded no bands and in which the internal standard did not amplify were discarded from the analysis. These reactions failed because of the presence of a *Taq* polymerase inhibitor in the sample.

### Statistical analyses

Statistical analyses were performed with Splus 6.2. Using Wilcoxon signed rank and rank sum tests, we tested whether mean and median TRF length differed among groups. Since TRF length distributions are typically positively skewed, median TRF length is theoretically a more robust and representative measure than the traditionally used mean TRF length. In all cases in which mean and median TRF length analysis yielded the same conclusion, only details for tests on the means are given. In the only case where the analysis on the means and medians yielded contradicting conclusions, both analyses are discussed. Telomerase data were distributed normally and were therefore analyzed using a two-tailed *t*-test. Skewness and kurtosis values were also distributed normally and were analyzed using two-way anovas (factors were caste and nest) with post-hoc Duncan's multiple range tests. Nest L128, which lacked a TRF length measurement for queens, was excluded from this analysis after verifying that this did not affect the outcome of the tests.
